# Impact of a universal testing and treatment intervention on HIV incidence in Zambia and South Africa: results of the HPTN 071 (PopART) community-randomized trial

**DOI:** 10.1056/NEJMoa1814556

**Published:** 2019-07-18

**Authors:** Richard J. Hayes, Deborah Donnell, Sian Floyd, Nomtha Mandla, Justin Bwalya, Kalpana Sabapathy, Blia Yang, Mwelwa Phiri, Ab Schaap, Susan H. Eshleman, Estelle Piwowar-Manning, Barry Kosloff, Anelet James, Timothy Skalland, Ethan Wilson, Lynda Emel, David Macleod, Rory Dunbar, Musonda Simwinga, Nozizwe Makola, Virginia Bond, Graeme Hoddinott, Ayana Moore, Sam Griffith, Nirupama Deshmane Sista, Sten H. Vermund, Wafaa El-Sadr, David N. Burns, James R. Hargreaves, Katharina Hauck, Christophe Fraser, Kwame Shanaube, Peter Bock, Nulda Beyers, Helen Ayles, Sarah Fidler

**Affiliations:** 1 London School of Hygiene and Tropical Medicine; 2 Fred Hutchinson Cancer Research Center; 3 Desmond Tutu TB Centre, Department of Paediatrics and Child Health, Faculty of Medicine and Health Sciences, Stellenbosch University; 4 Zambart; 5 London School of Hygiene and Tropical Medicine & Zambart; 6 Johns Hopkins University School of Medicine; 7 FHI 360; 8 Yale School of Public Health; 9 ICAP at Columbia University; 10 Division of AIDS, National Institute of Allergy and Infectious Diseases; 11 Imperial College London; 12 University of Oxford; 13 Imperial College London and Imperial College National Institute for Health Research Biomedical Research Centre

## Abstract

**Background:**

Universal testing and treatment (UTT) is a potential strategy to reduce HIV incidence, yet prior trial results are inconsistent. We report results from HPTN 071 (PopART), the largest HIV prevention trial to date.

**Methods:**

In this community-randomized trial (2013-18), 21 communities in Zambia and South Africa were randomized to Arm A (PopART intervention, universal antiretroviral therapy [ART]), Arm B (PopART intervention, ART per local guidelines), and Arm C (standard-of-care). The PopART intervention included home-based HIV-testing delivered by community workers who supported linkage-to-care, ART adherence, and other services. The primary outcome, HIV incidence between months 12-36, was measured in a Population Cohort (PC) of ~2,000 randomly-sampled adults/community aged 18-44y. Viral suppression (VS, <400 copies HIV RNA/ml) was measured in all HIV-positive PC participants at 24m.

**Results:**

The PC included 48,301 participants. Baseline HIV prevalence was similar across study arms (21%-22%). Between months 12-36, 553 incident HIV infections were observed over 39,702 person-years (py; 1.4/100py; women: 1.7/100py; men: 0.8/100py). Adjusted rate-ratios were A vs. C: 0.93 (95%CI: 0.74-1.18, p=0.51); B vs. C: 0.70 (95%CI: 0.55-0.88, p=0.006). At 24m, VS was 71.9% in Arm A; 67.5% in Arm B; and 60.2% in Arm C. ART coverage after 36m was 81% in Arm A and 80% in Arm B.

**Conclusions:**

The PopART intervention with ART per local guidelines reduced HIV incidence by 30%. The lack of effect with universal ART was surprising and inconsistent with VS data. This study provides evidence that UTT can reduce HIV incidence at population level.

**Trial registration:**

ClinicalTrials.gov NCT01900977

## Introduction

In 2017, ~37 million people were living with HIV worldwide, with 1.8 million new infections.^1^ HIV incidence is declining worldwide, but is unlikely to reach the UNAIDS target of <500,000 new infections by 2020.^2^ Steep reductions in incidence are needed to curb the HIV/AIDS epidemic.

Universal testing and treatment (UTT) has been proposed as an important component of HIV combination prevention programs.^3,4^ The HPTN 052 trial showed that early antiretroviral therapy (ART) initiation dramatically reduced HIV transmission among couples^5,6^, and the PARTNER study showed that viral suppression (<200 copies/ml) prevented HIV sexual transmission.^7,8^ Mathematical modeling predicted that HIV incidence would fall steeply if HIV testing were delivered throughout a population and ART initiated immediately after diagnosis.^9-11^ Early ART also confers individual health benefits.^12,13^ In 2015, the World Health Organization updated its guidelines recommending immediate ART for all HIV-positive individuals^14^, and UNAIDS proposed 90-90-90 HIV testing and treatment targets (by 2020: 90% of HIV-positive individuals should know their status; 90% of those individuals should be on ART; and 90% of those on ART should be virally suppressed).^15^

While there is compelling evidence supporting UTT for HIV prevention, it was not clear whether UTT could be implemented effectively at population level and impact HIV incidence. Four community-randomized trials (CRTs) in sub-Saharan Africa addressed these questions; two (TasP and SEARCH) reported no impact of UTT on HIV incidence; a third (Ya Tsie) reported a 30% reduction in incidence, of borderline statistical significance.^16-18^ The fourth study, HPTN 071 (PopART), is the largest HIV prevention trial ever conducted. Here, we present the primary results of HPTN 071 (PopART); we also describe the uptake of the PopART intervention and its impact on viral suppression.

## Methods

The study was designed by members of the Study Team with input from the sponsor, funders and government and non-governmental partners in Zambia and South Africa, listed in the Acknowledgements. The data were collected by staff of Zambart and the Desmond Tutu TB Centre in collaboration with LSHTM and the HPTN Statistical and Data Management Center. The data were analyzed by the analytic authors identified at the beginning of the manuscript who vouch for the integrity of the analysis. All authors vouch for the integrity of the data, contributed to the preparation and review of the manuscript and agreed to its publication. The initial draft was written by the first author. The sponsor required no agreements restricting access to the data or freedom to publish the study findings.

The study design has been described previously^19^ and is summarized below.

### Study population

HPTN 071 (PopART) was conducted between 2013-2018, in 21 urban/peri-urban communities in Zambia and Western Cape Province, South Africa (total population ~1 million; average ~50,000/community). Each community was the catchment population of a government clinic. Communities were arranged in seven triplets matched on geographical location and estimated HIV prevalence. Communities in each triplet were randomly allocated to three study arms in simultaneous public ceremonies. Restricted randomization was used to ensure balance across study arms on population size, baseline ART coverage and HIV prevalence.^19^

The three study arms are shown in Figure S1. Arm A communities received the PopART intervention (see below) with universal ART. Arm B communities received the PopART intervention with ART provided according to local guidelines. Arm C communities did not receive the PopART intervention, but received standard-of-care at government clinics, including HIV testing and ART offered according to local guidelines.

### Intervention

The PopART intervention, delivered to Arm A and B communities only, was a combination prevention package (Figure S2). Specially trained community health workers (Community HIV-care Providers, CHiPs) delivered services at annual household visits (see supplementary text). CHiPs worked in pairs, each pair responsible for ~500 households. Data collected by CHiPs were used primarily to support service delivery but also to evaluate intervention coverage.

At each visit, CHiPs offered HIV counseling and rapid testing, and provided support for linkage to care and ART adherence for HIV-positive clients. They referred uncircumcised HIV-negative men for voluntary medical male circumcision and HIV-positive pregnant women for antenatal care including prevention of mother-to-child HIV transmission. CHiPs also screened clients for symptoms of tuberculosis and sexually transmitted infections, with referral for diagnosis and treatment, and promoted and provided condoms.

In all 21 communities, HIV care and ART were provided at local government clinics. In Arm A, these clinics offered ART irrespective of CD4 count throughout the trial, with written consent for those initiating ART outside of local guidelines until universal ART became standard. In Arms B and C, the clinics provided ART initially at a CD4 threshold of 350 cells/ml, which increased to 500 cells/ml in 2014. Universal ART was offered from April 2016 (Zambia) and October 2016 (S Africa) (Figure S3).

### Outcome evaluation

The effect of the intervention on population-level HIV incidence was measured in a Population Cohort (PC) (enrolled December 2013-March 2015) that included one randomly-selected adult aged 18-44 years from a random sample of households in each community (Figure S4). PC participants were surveyed at baseline (PC0) and after 12, 24 and 36 months (PC12/PC24/PC36).

Because the original enrollment target (2,500 adults/community) was not reached in PC0, additional participants were enrolled at 12 months (PC12N) and in arms A and C only at 24 months (PC24N), excluding households sampled previously.

At each visit, PC participants were interviewed by a field research assistant (separate from the CHiPs) using a structured questionnaire that included collection of demographic, socio-economic and behavioral data, as well as data related to HIV prevention, diagnosis and treatment; data were collected electronically. Following the interview, blood was collected by a research nurse, who also offered HIV rapid testing to all PC participants.

The pre-defined primary study outcome was HIV incidence between PC12 and PC36, comparing Arm A and Arm B to Arm C. This approach provided one year to fully establish the study intervention before measuring study outcomes. Other outcomes reported here include viral suppression (VS, < 400 copies HIV RNA/ml) and the estimated coverage of HIV testing and ART based on CHiPs data from Arms A and B.

### Laboratory methods

Laboratory-based HIV testing was performed for all PC participants at all visits. Central laboratories in South Africa and Zambia performed a single 4^th^ generation HIV test. The HPTN Laboratory Center (LC, Baltimore, MD USA) performed additional testing to determine HIV status (see supplementary text). If seroconversion was confirmed, testing was performed to determine if the participant had acute infection at the prior visit. HIV viral load testing was performed at the HPTN LC for selected samples: all HIV-positive participants at PC24, and a random subset of ~75 HIV-positive participants per community at PC0, PC12 and PC36. HIV Viral load testing was performed using the Abbott Realtime HIV-1 assay (Abbott Molecular Inc, Des Plaines, Il) utilizing a <400 HIV RNA/ml threshold.

### Statistical considerations

Sample size calculations were informed by initial projections of intervention effect based on mathematical modeling^19,20^ which suggested that HIV incidence might be reduced by up to 60% in Arm A and 25% in Arm B, compared with Arm C. Assuming HIV incidence in Arm C of 1.0 to 1.5 per 100 person-years (py), a between-community coefficient of variation (k) within matched triplets of 0.15-0.20, 2,500 PC participants/community with 85% HIV-negative at baseline, and 25% loss to follow-up over three years, study power would exceed 75% or 85% for effects of 35% or 40%, respectively.

Analysis methods are described in detail in the Statistical Analysis Plan, completed before data unblinding.^21^ Briefly, HIV incidence was measured in PC participants who were HIV-negative at enrollment; HIV infection was assumed to occur at the mid-point between the last HIV-negative and the first HIV-positive sample, or at a visit where acute infection was identified. When timing of infection was unclear because of missed visits, the time of infection was estimated by imputation (see supplementary text and Table S1). For the primary outcome, statistical inference used a two-stage approach recommended for CRTs with <15 clusters/arm.^22,23^ At the first stage, Poisson regression on data from all three study arms was used to compute E, the expected number of events (incident HIV infections) in each community, after adjusting for age, sex and baseline HIV prevalence, assuming a null intervention effect. At the second stage, two-way analysis of variance was carried out on log(O/E) (log ratio-residuals), where O was the observed number of events in each community, with matched triplet and study arm as factors. The test statistic is the estimated difference in means of log(O/E) between study arms, with two-sided p-values and 95% confidence intervals (CI) computed using the t-distribution. The corresponding rate ratios and 95%CI for the comparison of Arms A and C, and Arms B and C, were calculated with exponentiation. Similar methods were used for the analysis of viral suppression, except that logistic regression was used at the first stage without adjustment for HIV prevalence. The robustness of the above analyses was assessed using a permutation test based on the restricted randomization scheme.

Because the analysis plan did not include a method for controlling type I error when conducting treatment comparisons for subgroup and *post-hoc* analyses, treatment effects are reported with point estimates and 95% confidence intervals (which have not been adjusted for multiplicity and should not be used to infer treatment effects).

In Arms A and B, CHiPs data were used to estimate the proportion of HIV-positive community members who knew their HIV status and were on ART, using methods and assumptions described in supplementary text.

### Ethical considerations

PC participants provided written informed consent before enrollment. Community members visited by CHiPs provided verbal consent for participation in the intervention and data collection. In Arm A, clinic patients provided written informed consent when ART was initiated outside of prevailing local guidelines (2013-2016).

Ethical approval for the study was granted by ethics committees at the London School of Hygiene and Tropical Medicine, University of Zambia, and Stellenbosch University.

## Results

### Enrollment and follow-up

**Figure 1:**
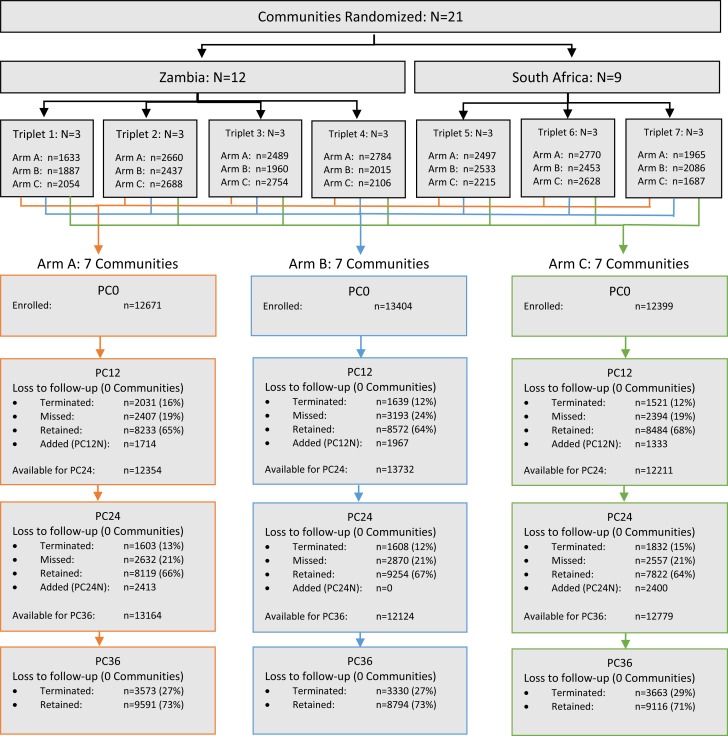
CONSORT diagram showing enrollment and follow-up of the Population Cohort. HPTN 071 (PopART) included 21 communities that were matched in seven sets of three communities each; the three communities in each triplet were randomized to Study Arms A, B, and C. The purpose of the Population Cohort (PC) was to enrol and follow a representative sample of residents to assess the impact of the PopART intervention on HIV incidence and viral suppression. Participants in the PC were enrolled from randomly selected households in the community; with one member aged 18-44 selected at random for eligibility assessment. The diagram shows the number of participants enrolled at the start of the study (PC0). Additional participants were enrolled in PC12N in communities with fewer than 2000 PC0 participants; additional participants were enrolled in Arms A and C in PC24N to preserve power for this comparison. The status of participants at each survey year (PC12, PC24, PC36) is reported. Individuals who missed yearly follow-up visits were eligible for subsequent annual surveys, individuals who were terminated were not. The percentage retained is the proportion of participants who completed a visit amongst those eligible for the visit.

The CONSORT diagram (Figure 1) shows the enrollment and follow-up of PC participants; 38,474 adults were enrolled at baseline (PC0), with 5,014 and 4,813 additional enrollments at PC12N and PC24N, respectively (total enrolled: 48,301).

At PC12, and again at PC24, 13% of PC participants were terminated from the study, most because of confirmed permanent relocation out of the study community (Table S2), and were censored from further observation; ~75% of remaining participants completed each visit. The final survey (PC36) reached 72% of eligible participants. Retention was similar across study arms at PC36 (73%, 73% and 71% in Arms A, B and C, respectively).

### Baseline comparisons

More women (71%) than men (29%) were enrolled in PC0, with 40% of participants aged <24 years (Table 1). Socio-demographic and behavioral characteristics were similar across study arms. Approximately 17% of men reported having undergone medical circumcision.

Baseline HIV prevalence was 22% (women: 26%, men: 12%) and baseline *Herpes simplex* virus type-2 (HSV-2) prevalence was 46% (women: 54%, men: 24%). The prevalence of both infections was similar across study arms (HIV: 21% Arm A, 21% Arm B, 22% Arm C; HSV-2: 46% Arm A; 46% Arm B, 45% Arm C). Reported ART coverage was slightly higher in Arm B (33% Arm A, 41% Arm B, 35% Arm C), but the proportion of HIV-positive participants with VS at PC0 was similar across study arms (56% Arm A, 57% Arm B, 54% Arm C).

**Table 1:** Characteristics of population cohort at baseline (PC0) The table shows baseline characteristics of the PC in the three study arms. The table is restricted to PC participants enrolled at PC0. Data are pooled across all seven communities in each study arm.

Baseline Variable	Arm A	Arm B	Arm C
**Total enrolled (PC0)**	12671	13404	12399
**Sex**			
Male	3595 (28%)	3906 (29%)	3701 (30%)
Female	9042 (72%)	9458 (71%)	8639 (70%)
Missing	34	40	59
**Age (years)**			
18-24	5065 (40%)	5179 (39%)	4981 (40%)
25-34	4928 (39%)	5170 (39%)	4688 (38%)
35-44	2643 (21%)	3015 (23%)	2667 (22%)
Missing	35	40	63
**Marital status**			
Married/living as married	5363 (43%)	5210 (39%)	4693 (38%)
Never married	6292 (50%)	6923 (52%)	6644 (54%)
Divorced/separated	708 (6%)	892 (7%)	656 (5%)
Widowed	197 (2%)	208 (2%)	206 (2%)
Missing	111	171	200
**Nights spent away from community (past 3m)**			
None	11623 (94%)	10650 (87%)	10864 (89%)
1-7	556 (4%)	890 (7%)	886 (7%)
8-14	97 (1%)	228 (2%)	178 (1%)
15+	149 (1%)	418 (3%)	245 (2%)
Missing	246	1218	226
**Number of sexual partners (past 12m)**			
0	3160 (27%)	4266 (33%)	3188 (27%)
1	8032 (68%)	7663 (60%)	7913 (66%)
2-4	496 (4%)	753 (6%)	722 (6%)
5+	70 (1%)	121 (1%)	81 (1%)
Missing	913	601	495
**Male circumcision (self-report)^1^**			
Not circumcised	1725 (51%)	1974 (53%)	1904 (55%)
Medical	567 (17%)	613 (16%)	646 (19%)
Traditional	1113 (33%)	1171 (31%)	895 (26%)
Missing	190	148	256
**ART coverage^2^**			
Yes	788 (33%)	1048 (41%)	878 (35%)
No	1587 (67%)	1534 (59%)	1648 (65%)
Missing	208	152	161
**HIV prevalence**			
Negative	9594 (79%)	10235 (79%)	9301 (78%)
Positive	2583 (21%)	2734 (21%)	2687 (22%)
Not determined^3^	494	435	411
**HSV-2 prevalence**			
Negative	6506 (53%)	7005 (54%)	6585 (55%)
Positive	5667 (46%)	5959 (46%)	5357 (45%)
Indeterminate	64 (1%)	55 (<1%)	74 (1%)
Not determined^4^	434	385	383
**HIV viral suppression^5^**			
Yes	295 (56%)	300 (57%)	267 (54%)
No	228 (44%)	225 (43%)	227 (46%)

1 For male circumcision the denominator is the number of men.

2 ART coverage is the proportion of HIV-positive participants self-reporting current ART use. The denominator is the number of HIV-positive participants.

3 HIV status not determined occurred when a participant did not consent to specimen collection, no sample was available or when lab testing did not result in a determination of infection status.

4 HSV-2 status not determined occurred when a participant did not consent to specimen collection, or no sample was available.

5 Viral suppression was assessed in a random sample of ~75 HIV-positive participants per community in PC0.

Missing data are excluded from % calculations which are based on data pooled across communities.

Baseline comparisons between arms include only PC0 participants as this best represents the balance between arms in the communities prior to the delivery of the intervention.

### Impact of the intervention on HIV incidence

**Figure 2:**
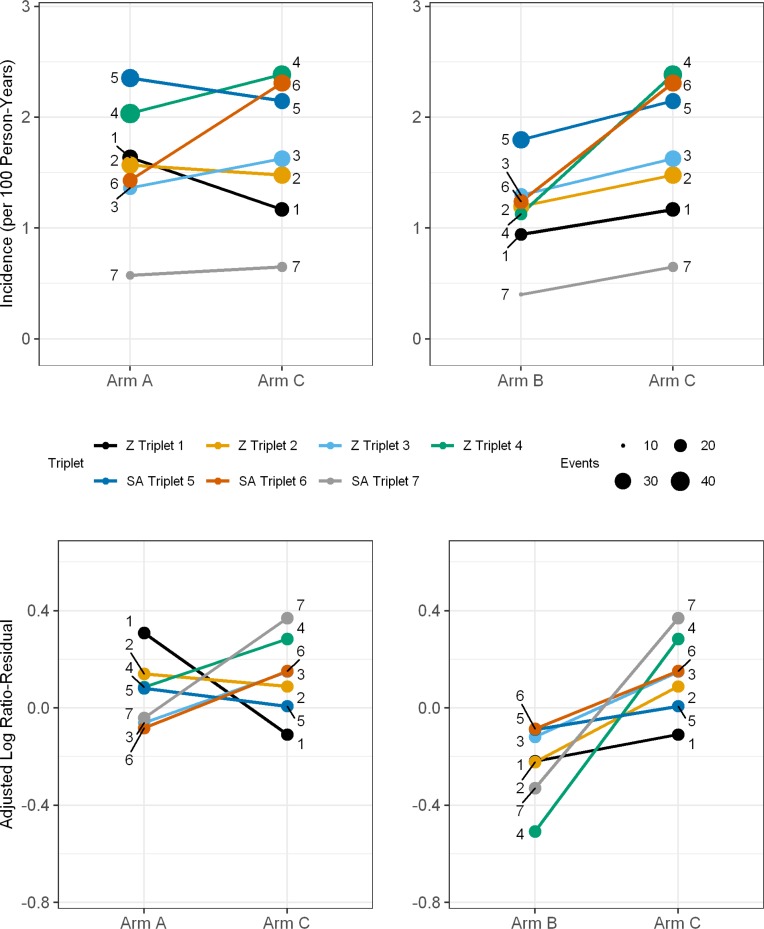
Estimates of HIV incidence and log ratio-residuals for the seven study triplets. The plots show estimates of HIV incidence (plotted per 100 person-years upper panels) and log ratio-residuals (observed/expected HIV infections adjusted for age, sex and baseline HIV prevalence, lower panels) for Arm A vs. Arm C and Arm B vs. Arm C. Data are shown for the study period included in the primary endpoint analysis (PC12 to PC36). Colored lines represent each of the seven triplets (numbered 1 to 7). For HIV incidence, the size of the colored dot at the end of each line represents the number of events contributing to the incidence estimate for each community. Abbreviations: Z: Zambia; SA: South Africa.

Estimated effects of the intervention on HIV incidence are shown in Table 2 and Figure 2.

**Table 2:** Effect of PopART intervention on HIV incidence and HIV viral suppression The table shows the HIV incidence rate between PC12 and PC36 and proportion of HIV-positive participants with viral suppression at PC24 for each triplet and overall, and for men and women, in each study arm. The table also shows the unadjusted and adjusted rate ratios for incidence and viral suppression overall, and for men and women. Viral suppression was defined as HIV viral load <400 copies/mL.

Outcome	Arm A	Arm B	Arm C
**HIV incidence rate (PC12-PC36)**	*No. of events/total person-years (rate per 100 person-years)^1^*		
Triplet 1	28/1687 (1.64)	19/1979 (0.94)	24/2054 (1.17)
Triplet 2	33/2086 (1.57)	29/2408 (1.20)	33/2262 (1.48)
Triplet 3	23/1695 (1.36)	22/1687 (1.30)	29/1811 (1.63)
Triplet 4	41/2013 (2.04)	19/1698 (1.13)	37/1561 (2.39)
Triplet 5	36/1507 (2.35)	33/1811 (1.80)	28/1304 (2.15)
Triplet 6	26/1808 (1.43)	26/2078 (1.24)	32/1375 (2.31)
Triplet 7	13/2195 (0.57)	10/2488 (0.40)	14/2195 (0.59)
Overall IR^2^	198/12990 (1.45)	157/14149 (1.06)	198/12563 (1.55)
	**Arm A vs Arm C**	**Arm B vs Arm C**	
Unadjusted rate ratio (95% CI)	0.94 (0.77, 1.15)	0.68 (0.56, 0.84)	1
P value^3^	0.505	0.002	
Adjusted rate ratio^4^ (95% CI)	0.93 (0.74, 1.18)	0.70 (0.55, 0.88)	1
P value^5^	0.509	0.006	
**Men**			
Overall IR^2^	36/3766 (0.77)	23/4301 (0.45)	39/4115 (0.92)
Adjusted rate ratio^4^ (95% CI)	0.88 (0.41, 1.88)	0.52 (0.24, 1.12)	1
Women			
Overall IR^2^	162/9225 (1.71)	134/9848 (1.26)	159/8448 (1.79)
Adjusted rate ratio^4^ (95% CI)	0.96 (0.72, 1.28)	0.73 (0.55, 0.97)	1
P value for interaction by sex	0.794	0.401	
	**Arm A**	**Arm B**	**Arm C**
**Viral suppression (PC24)**	*No. VS/total no. HIV-positive (%)*		
Triplet 1	140/175 (80.0%)	183/244 (75.0%)	212/290 (73.1%)
Triplet 2	204/311 (65.6%)	276/371 (74.4%)	179/271 (66.1 %)
Triplet 3	225/295 (76.3%)	177/255 (69.4%)	174/284 (61.3%)
Triplet 4	356/518 (68.7%)	219/324 (67.6%)	354/476 (74.4%)
Triplet 5	270/389 (69.4%)	275/381 (72.2%)	211/315 (67.0%)
Triplet 6	250/355 (70.4%)	126/202 (62.4%)	338/506 (66.8%)
Triplet 7	86/116 (74.1%)	62/114 (54.4%)	12/41 (29.3%)
Overall prevalence^6^	1531/2159 (71.9%)	1318/1891 (67.5%)	1480/2183 (60.2%)
	**Arm A vs Arm C**	**Arm B vs Arm C**	
Unadjusted VS prevalence ratio (95% CI)	1.19 (0.97, 1.47)	1.12 (0.91, 1.38)	1
P value^3^	0.090	0.258	
Adjusted VS prevalence ratio^7^ (95% CI)	1.16 (0.99, 1.36)	1.08 (0.92, 1.27)	1
P value^3^	0.071	0.297	
Men			
Overall prevalence^6^	183/294 (63.0%)	153/244 (60.8%)	179/330 (40.0%)
Adjusted VS prevalence ratio^7^ (95% CI)	1.46 (0.86, 2.48)	1.41 (0.83, 2.41)	1
Women			
Overall prevalence^6^	1348/1865 (73.3%)	1165/1647 (68.4%)	1301/1853 (65.8%)
Adjusted VS prevalence ratio^7^ (95% CI)	1.10 (1.00, 1.22)	1.03 (0.93, 1.13)	1
P value for interaction by sex	0.220	0.164	

Abbreviations: IR = incidence rate; VS = viral suppression (<400 copies/mL).

1 Imputation was used to estimate missing timing of HIV infection in seroconverting participants who missed PC12 or PC24 (See supplementary material)

2 Overall IR is geometric mean of individual community IR

3 P-value compared to t-distribution with 12 degrees of freedom.

4 Adjusted for age, sex, baseline HIV prevalence

5 P-value compared to t-distribution with 11 degrees of freedom.

6 Overall prevalence is geometric mean of individual community proportions with viral suppression

7 Adjusted for age, sex

Between PC12 and PC36 (primary outcome), 553 incident HIV infections were observed during 39,702py follow-up (1.4/100py; women: 1.7/100py; men: 0.8/100py). Incidence in Arm C (geometric mean across communities) was 1.6/100py overall (Table 2). Incidence in Arm A was 1.5/100py; the adjusted rate ratio (AdjRR) compared with Arm C was 0.93 (95%CI: 0.74-1.18, p=0.51). Incidence in Arm B was 1.1/100py; the AdjRR compared with Arm C was 0.70 (95%CI: 0.55-0.88, p=0.006). HIV incidence was lower in Arm B vs. Arm C in all seven matched triplets, while incidence was lower in Arm A vs. Arm C in only four triplets (Figure 2). A permutation test based on the restricted randomization scheme showed even stronger evidence of an effect in Arm B vs. Arm C (p=0.001), but not in Arm A vs. Arm C (p=0.48). The findings were essentially similar when the analysis was restricted to PC participants enrolled at PC0 (Table S6).

In Arm B vs. Arm C, subgroup analyses by sex (Table 2) and age and sex (Table S3) showed a greater effect on HIV incidence in men (AdjRR: 0.52, 95%CI: 0.24-1.12) than women (AdjRR: 0.73, 95%CI: 0.55-0.97), although this difference in effect could have occurred by chance (p for interaction = 0.40); there was also evidence of a greater effect in older participants (aged 25+; AdjRR: 0.58, 95%CI: 0.43-0.76) than younger participants (18-24y; AdjRR: 0.92, 95%CI: 0.70-1.20; p for interaction = 0.044). HIV incidence and estimated effects for individual years of follow-up, and for the entire study period (PC0-PC36) are shown in Tables S4 and S5. HIV incidence decreased in Arm C by 12% (95%CI:0%-23%) per year (Figure S5).

### Impact of the intervention on viral suppression

Proportions of HIV-positive PC24 participants with VS were 71.9% in Arm A, 67.5% in Arm B and 60.2% in Arm C (Table 2). The adjusted VS prevalence ratios were 1.16 (95%CI: 0.99-1.36, p=0.07) for Arm A vs. Arm C and 1.08 (95%CI: 0.92-1.27, p=0.30) for Arm B vs. Arm C. In Arms A and B, VS at PC24 was higher in women than in men, and considerably higher in those aged ≥25 years than those aged 18-24 years (Table 2 and Table S7). VS in Arms A and B increased steeply from ~55% at PC0 to ~75% at PC36 (Table S8). VS in participants who self-reported ART use was consistently high in Arms A and B (86-91%, Table S9).

### Coverage of the intervention

**Figure 3:**
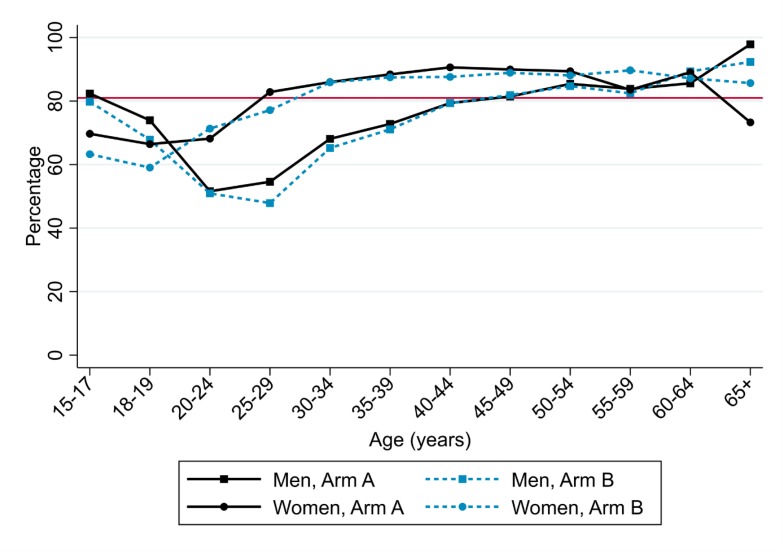
Estimated ART coverage at the end of the study, by age and sex and study arm; estimated from the CHiPs data and extrapolated to total population aged ≥15 years The plot shows estimated ART coverage among the total population aged ≥15 years in Arm A and B communities at the end of the study, by sex, age-group and study arm. Coverage estimates are shown in black solid lines for Arm A and in blue dashed lines for Arm B. Lines for men are shown with a square symbol, and for women with a circle symbol. The UNAIDS 90-90-90 target for ART coverage (81%) is shown in red. The estimated number of HIV-positive men who were resident in the community at the time that CHIPs first visited their household during the third (and last) annual round of intervention, and remained resident in the study community at the end of the study, was 8,388 in Arm A and 8,948 in Arm B, and the estimated number of HIV-positive women was 15,936 in Arm A and 17,586 in Arm B. The estimated number of HIV-positive men on ART was 6,286 in Arm A and 6,378 in Arm B, and the estimated number of HIV-positive women on ART was 13,600 in Arm A and 14,481 in Arm B.

Based on CHiPs data, the estimated proportions of all HIV-positive adults who were on ART at the end of the study were 81% in Arm A and 80% in Arm B (Table S10). Figure 3 shows estimated ART coverage by age and sex, indicating similar coverage in Arms A and B, lower coverage in men than women, and lower coverage among younger compared with older individuals. ART coverage was also similar in Arms A and B in most triplets (Figure S6).

## Discussion

This study provides evidence that UTT can reduce HIV incidence at population level. In Arm B, HIV incidence was reduced by 30% compared to the standard-of-care control arm; surprisingly, there was no evidence of such an effect in Arm A.

The Arm B effect was consistent with pre-study model projections and was observed in both countries.^20^ Reduction in incidence was seen in all seven matched triplets in Arm B; this effect was very unlikely to have occurred by chance. UTT is hypothesized to reduce HIV transmission by increasing the proportion of HIV-positive community members who know their HIV status, the proportion of those individuals who are on ART, and the proportion of those on ART who are virally suppressed. Data from this study indicate that the UNAIDS 90-90-90 targets were achieved by the end of the 3-year intervention in both Arm A and B communities. High levels of VS were observed among HIV-positive PC participants after 24m (~72%, increased from the baseline level of ~55%). This corresponds to a ~35% drop in the proportion of HIV-positive participants not virally suppressed, from ~45% to ~28%, consistent with the observed 30% reduction in HIV incidence in Arm B. The greater reduction in HIV incidence among men likely reflects greater uptake of the intervention and higher VS in women (thus protecting their male partners); a similar explanation applies for higher effectiveness in those aged over 25 years.

There are several possible explanations for the lack of an effect on HIV incidence when the PopART intervention was combined with universal ART (Arm A vs. Arm C). First, written informed consent was required for initiation of ART outside local guidelines from the start of the trial until 2016 (see supplementary text). This requirement for “research consent” may inadvertently have discouraged ART initiation, although this is not supported by data that show similar ART coverage and VS in Arms A and B. Second, wide-scale ART delivery in Arm A may have led to sexual disinhibition or de-emphasis of primary prevention messaging by CHiPs, offsetting the observed increase in VS. Data on self-reported risk behaviors do not support this hypothesis; further analyses are planned once data on HSV-2 seroconversion (a proxy for sexual risk behavior) become available. Third, while the three study arms appeared well matched with respect to baseline data, there may have been unrecognized differences across triplets in socio-demographic or other factors, such as mobility and migration resulting in exposure to HIV-positive partners from other communities.

While these urban communities had high mobility, analysis of available data do not suggest any appreciable differences in migration across study arms. Further analyses of qualitative and quantitative data from the study communities, and data from an ongoing phylogenetic study, may shed light on the unexpected Arm A result.

Strengths of the study included the large sample size, enrollment of a randomly-sampled cohort to measure HIV incidence and VS at community level, delivery of ART through routine services at government clinics, the availability of extensive process data used to improve and refine delivery of the intervention, and strong community engagement. While our study communities were not chosen to be representative of Zambia or South Africa as a whole, conduct of the study in large urban communities with high rates of mobility should increase the generalizability of the findings to other urban areas of Southern Africa with generalized HIV epidemics.

A limitation of the study was the relatively small number of randomized communities (seven/arm). The difference in observed effects in Arm A vs C and B vs C may thus be a chance finding, given the similar levels of ART coverage and VS in Arms A and B, and the similar nature of the Arm A and B interventions during most of the primary analysis period. To evaluate the overall effect of the PopART intervention vs standard of care, we therefore conducted a *post-hoc* analysis combining Arms A and B and found an estimated rate ratio of 0.81 (95%CI:0.66-0.99) compared with Arm C, consistent with a 20% reduction in incidence. Another limitation is that data on uptake of interventions among HIV-positive participants in the PC may be subject to a Hawthorne effect, because participants had regular contact with research staff offering HIV testing and providing referral to care. We would expect the Hawthorne effect to be greatest in Arm C, where PC participants did not have access to CHiP services for testing and referral. Thus, for uptake estimates we rely mainly on intervention data, which were only available from Arm A and B communities. Lastly, men were under-represented in the PC, and a substantial number of PC participants moved out of the community during follow-up and were censored from further observation. Thus, we cannot rule out selection bias although there was no evidence that these factors differed between study arms. Since men were under-represented in the PC, and a greater effect of the intervention was observed in men, the population-level effect may have been underestimated.

The results of HPTN 071 (PopART) are consistent with programmatic and survey data^24-27^, and should be considered alongside those of the other three trials that measured the effect of UTT on HIV incidence in Africa, all of which were smaller and undertaken in largely rural communities. The TasP trial^16^ found no effect on HIV incidence, which may have reflected the similar HIV testing services provided in the intervention and control arms, with low levels of ART coverage in both arms. The SEARCH trial^17^ also found no effect on HIV incidence, which may have reflected intensive baseline community-based HIV testing in both intervention and control arms. The Ya Tsie trial^18^ observed a 30% reduction in incidence, which was of borderline statistical significance given the relatively small numbers of HIV seroconversion events.

Our finding of a 20-30% reduction in HIV incidence at population level was measured against a background of decreasing incidence in Arm C, possibly attributable to gradually increasing coverage of ART in the general population. This indicates that combination prevention including UTT can make a substantial contribution to HIV epidemic control. Importantly, the effects seen in our study, Ya Tsie study and others^28^ were achieved by delivering intensive household-based HIV-testing services; this may have played a more important role than changes in ART guidelines. The universal “test” component of a “test-and-treat” strategy is vital, as is continued attention to primary HIV prevention interventions.^29,30^ Results from planned cost-effectiveness and modeling studies will provide information on the value-for-money and long-term impact of such strategies which will help to inform policy and practice. ART coverage data from HPTN 071 (PopART), like data from other studies, draws special attention to the challenges in achieving ART coverage targets in young people, men, and communities with high mobility.^31-33^ If HIV transmission is concentrated in these subgroups, impact of UTT on HIV transmission may be compromised. Special efforts will be needed to address these coverage gaps to realize the full impact of UTT on HIV epidemic control.
