# Supplementary material for: Impact of a universal testing and treatment intervention on HIV incidence in Zambia and South Africa: results of the HPTN 071 (PopART) community-randomized trial
Source: N Engl J Med. Author manuscript; Available in PMC 2019 Jul 18. (PMC6587177; doi:10.1056/NEJMoa1814556)
Supplement: Supplementary file 1 [file NEJM-2019-1814556-s1.pdf]

Supplementary Material

Contents

TEXT

Detailed description of the household intervention delivered to Arms A and B, ART delivery during the study period and Arm C activities 3

Identification of incident HIV infections 7

Imputation methods 8

Estimation of the number of HIV-positive individuals in the population, ART coverage, and coverage against the first and second of the UNAIDS 90-90-90 targets, at the end of the study 9

Figure S1 - Design of the HPTN 071 (PopART) study, a 3-arm cluster-randomized trial in 21 communities 11

Figure S2 - PopART combination prevention package and household intervention 13

Figure S3 - Timeline of HPTN 071 (PopART) study showing annual intervention rounds (R1-R3), Population Cohort survey rounds (PC0-PC36), the primary analysis period and changes in ART initiation thresholds by country 15

Figure S4 - Screening and enrollment in PC0 17

Figure S5 - HIV incidence over study years in Arm C communities 18

Figure S6 - Estimated ART coverage at the end of the study, by triplet and study arm; estimated from the CHiPs data and extrapolated to total population aged > 15 years 19

Table S1 - Number of eligible seroconverters and participants with missed visits affected by imputation 20

Table S2 - Reasons for Termination by arm 21

Table S3 - Effect of PopART intervention on HIV Incidence: subgroup analyses by age and sex. 23

Table S4 - Effect of PopART intervention on HIV Incidence for each study year: PC0-PC12, PC12-PC24, PC24-36. 25

Table S5 - Effect of PopART intervention on HIV incidence for entire study period (PC0-PC36). 26

Table S6- Effect of PopART intervention on HIV incidence assessed in participants enrolled at PC0. Number of events and person years include imputation for seroconverters who missed PC12 and PC24 visits. 28

Table S7 - Effect of PopART intervention on viral suppression at PC24: subgroup analyses by age and sex 29

Table S8 - Viral suppression in HIV-positive PC participants at each study year. 30

Table S9 - Viral suppression at PC24 in HIV-positive PC participants who self-report current ART use. 31

Table S10 - ART coverage, and coverage against the first two of the UNAIDS 90-90-90 targets, at the end of the study, by study arm; estimated from CHiP data and extrapolated to total population aged ≥15 years 32

REFERENCES 34

Detailed description of the household intervention delivered to Arms A and B, ART delivery during the study period and Arm C activities

The (PopART) CHiPs intervention

The PopART HIV combination prevention package included annual rounds of household visits by Community HIV-care Providers (CHiPs) in the Arm A and Arm B communities, attempting to reach every household in every community at least once per round for the duration of the trial (November 2013-December 2017). At each “annual round” household visit, a team of two CHiPs staff approached a household, requested verbal consent to enter and explain the intervention being offered, and then, with permission, provided HIV education and prevention information, offered HIV rapid testing, offered referral of HIV-positive (HIV+) individuals to the local government healthcare facilities for HIV care/ART, offered referral to medical male circumcision services for HIV-negative men, offered referral to antenatal care for HIV+pregnant women, offered referral for reproductive health and family planning services, provided tuberculosis symptom screening followed by collection of sputum samples and referral to the health facility for those diagnosed with TB, offered sexually-transmitted-infection symptom screening followed by referral of those with symptoms to the health facility for diagnosis, and provided condoms. Following the initial household visit in an annual round, CHiPs would return to a household during the round to offer the intervention to those not available at a prior visit, to encourage linkage to care for those previously referred for HIV services, and to support retention for those on ART.

Before the start of the PopART intervention, each of the intervention communities was divided into “zones” with on average ~500 households in each zone. Each pair of CHiPs was responsible for providing the intervention to all households in one zone. In the first “annual round” (November 2013 to June 2015), CHiPs attempted to contact all adults (≥18 years) in their zone at least once. The CHiPs’ focus broadened in round 2 (June 2015 to October 2016) in that from early in this round (01

October 2015), CHiPs also attempted to contact all those aged 15–17 years at least once. This effort continued throughout the third round (October 2016- December 2017). Although the focus for the CHiPs was on individuals in the age ranges described above, the intervention was offered to all household members irrespective of age, and HIV testing for children was offered with parental or guardian consent, in accordance with national guidelines for age of consent for HIV testing.

During Round 2, and continued in Round 3, additional strategies were implemented to try to increase the percentage of men who were contacted by the CHiPs, and to facilitate more rapid linkage to HIV care among HIV-positive individuals. Depending on the community, these strategies sometimes included focused, short-term campaigns ("zonal campaigns") in a particular CHiP zone, or conduct of HIV testing drives at venues outside the household setting, such as taxi ranks or markets. Having CHiPs work shifts early or late in the day and on weekends was an effective strategy. To increase the reach to men and youth, male health fairs ("Man-Up" campaigns) and work place testing and HIV awareness campaigns were delivered. To encourage youth to link to care and access prevention materials and condoms, special youth-friendly corners were created in healthcare facilities. There was a greater focus on linkage to care generally in Rounds 2 and 3 compared with Round 1, with targeted follow-up of individuals who had been referred but not yet linked to care, increased coordination between the CHiPs and the clinic to facilitate linkage, and clinic improvements. A separate manuscript focused on linkage to care in the PopART intervention has been published.[1] At the end of Round 3, there was particular emphasis on linking to care any HIV-positive individuals not yet on ART.

The PopART intervention employed 740 CHiPs in total. In each annual round, CHiPs visited ~150,000 households, enumerating (listing) ~380,000 household members aged $\geq$15 years and providing the intervention to ~260,000 aged $\geq$15.

ART delivery during the study period

In all communities, irrespective of study arm, ART was delivered through local government healthcare facilities' routine ART clinics. ART drugs used were those of standard first and where needed second line therapy; comprising tenofovir, lamivudine and efavirenz (600mg od) for all HIV+ individuals eligible for first line therapy and lopinavir/ritonavir with zidovudine and 3TC for second line therapy, in accordance with national procurement procedures, and according to national ART guidelines.[2-7] Prior to the start of HPTN 071 (PopART), the trial ensured procurement of ART medicines necessary to ensure that drug stocks were always available in all study ART healthcare facilities for the duration of the trial. At the beginning of the trial, for individuals attending Arm A clinics whose CD4 count was above the threshold for ART initiation per national guidelines (see Figure S3), written informed consent was taken by PopART research nurses to initiate ART. By the end of 2016, all healthcare facilities in all 21 communities offered ART irrespective of CD4 count at initiation, and therefore written consent was no longer required in Arm A. Retention in care and ART monitoring on therapy was done in accordance with national guidelines and through routine government facilities.

Arm C activities

In the seven Arm C communities, the study provided no community-based HIV testing. In some Arm C communities, there were times when other organizations or programs did provide mobile HIV testing campaigns, so some non-facility based HIV testing did take place during the study period. In all Arm C communities, any individual wishing to test for HIV could do so through routine government HIV testing services, with ART initiation according to national guidelines. In preparation for the study, additional training focused on HIV care and treatment was provided to the staff at all Arm C clinics to ensure that the care provided during the study period met national standards. Drug stores were supplemented to ensure no drug-stock outs occurred during the trial period. After ART

treatment guidelines shifted to universal ART (2016) in Zambia and South Africa, ART was offered to all HIV+ individuals attending an Arm C clinic. The key difference for a community member living in an Arm C community compared with those living in an Arm A or B community was the absence of the household intervention provided by CHiPs.

Identification of incident HIV infections

In HPTN 071 (PopART), HIV incidence was determined based on laboratory testing of samples collected from participants in the Population Cohort (PC). Details of the procedures used for HIV testing and the results of that testing will be presented elsewhere. A brief summary is provided below.

HIV status was determined for all PC participants at all study visits, using samples collected during household visits. Centralized laboratories in Zambia and South Africa performed a single HIV screening assay. Additional HIV testing was performed at the HPTN Laboratory Center (LC) using pre-specified testing algorithms for quality control and to identify incident HIV infections. Incident HIV infections were confirmed by documenting a change in HIV status from HIV uninfected to HIV infected. HIV infection was confirmed by detection of HIV RNA and/or detection of HIV antibodies using a discriminatory HIV test. If incident infection was documented, an HIV RNA test was used to determine if a participant had acute HIV infection at the visit prior to seroconversion; if test results were consistent with acute HIV infection, the visit was considered to be the first HIV-positive visit. HIV status was not assigned at any visit for cases where HIV testing indicated a laboratory error (possible sample mixup or testing/data error) or if HIV status across visits could not be established because of missing and/or inconsistent test results. Selected cases, including cases with incomplete or discordant test results and cases with an acute HIV infection visit, were reviewed by an External Adjudication Committee.

Imputation methods

Imputation methods were used in the estimation of primary effectiveness for HIV incidence. Imputation was used for participants who had eligible follow-up for the primary effectiveness period (PC12-PC36) but missed a visit or did not have an HIV status available at PC12 or PC24. For participants who were HIV-negative following a missed visit, HIV status was imputed as HIV-negative at the missed visit. For participants with missed visits who seroconverted (e.g. were HIV-negative at PC0, missed PC12 and were HIV-positive at PC24), hot deck imputation (a form of bootstrap) was used to impute HIV status at the missed visit. For each seroconverter, HIV status at the missed visit was sampled at random from the pool of seroconverters over the same time period, matching gender and community. The timing of the visit was imputed using mean imputation for each community: the visit date was estimated between observed visits, using the mean proportional timing of follow-up for participants with completed visits.

Table S1 shows the number of PC participants and seroconverters with missing information on timing of HIV infection because of missing visits at PC12 and/or PC24. Approximately 50% of the person years in PC0-PC24; 67% of the person years in PC0-PC36 and 100% of the person years in PC12-36 were included in the primary analysis using imputation.

Twenty imputation datasets were created, and the primary analysis results computed for each imputation dataset. The primary endpoint estimate was the mean of the 20 imputation estimates and 95% confidence intervals were constructed using standard imputation methods.

Estimation of the number of HIV-positive individuals in the population, ART coverage, and coverage against the first and second of the UNAIDS 90-90-90 targets, at the end of the study

Methods for estimating the number of HIV-positive individuals, coverage against the first and second 90 targets, and ART coverage, have been described previously[8,9], and are summarized here.

Among *individuals who participated in the third and last "annual round" of intervention*, we estimated the number of HIV-positive individuals as the number who were "known HIV-positive" to the CHiPs (because they self-reported they were HIV-positive, or tested HIV-positive) plus an estimated number among those whose HIV status was not known to CHiPs (because they did not self-report HIV-positive, did not test with CHiPs, and did not report an HIV-negative test result in the previous 3 months). Among those whose HIV status was unknown to CHiPs, we assumed that HIV prevalence was the same as among those who tested for HIV. We then calculated:

1) the proportion who knew their HIV-positive status *immediately following the annual round* as the total who were known by the CHiPs to be HIV-positive following the annual round visit, divided by the estimated number of HIV-positive individuals;
2) the proportion who were on ART *by the end of the study,* among those who knew their HIV-positive status, as the total who self-reported they were on ART at the last CHiP visit made during the study, divided by the number who were known by the CHiPs to be HIV-positive and who remained resident in the same zone of the community according to the last information collected during the study.

We extrapolated to the total population by assuming that HIV prevalence was the same in non-participants and participants, and that knowledge of HIV-positive status and ART uptake among non-participants was the same as among participants *immediately before* the third annual round. Following this, we estimated coverage against the "first 90" target (percentage of HIV-positive

individuals who know their HIV-positive status) and coverage against the “second 90” target (percentage of individuals who are on ART, among all HIV-positive individuals who know their HIV-positive status) in the total population.

ART coverage was estimated as the product of estimated coverage against the first 90 target multiplied by estimated coverage against the second 90 target.

All estimates were calculated within strata defined by community of residence, sex, and age group; for Zambia (but not South Africa), estimates were also stratified on participation, residency, and HIV status in previous rounds of intervention

Figure S1 - Design of the HPTN 071 (PopART) study, a 3-arm cluster-randomized trial in 21 communities

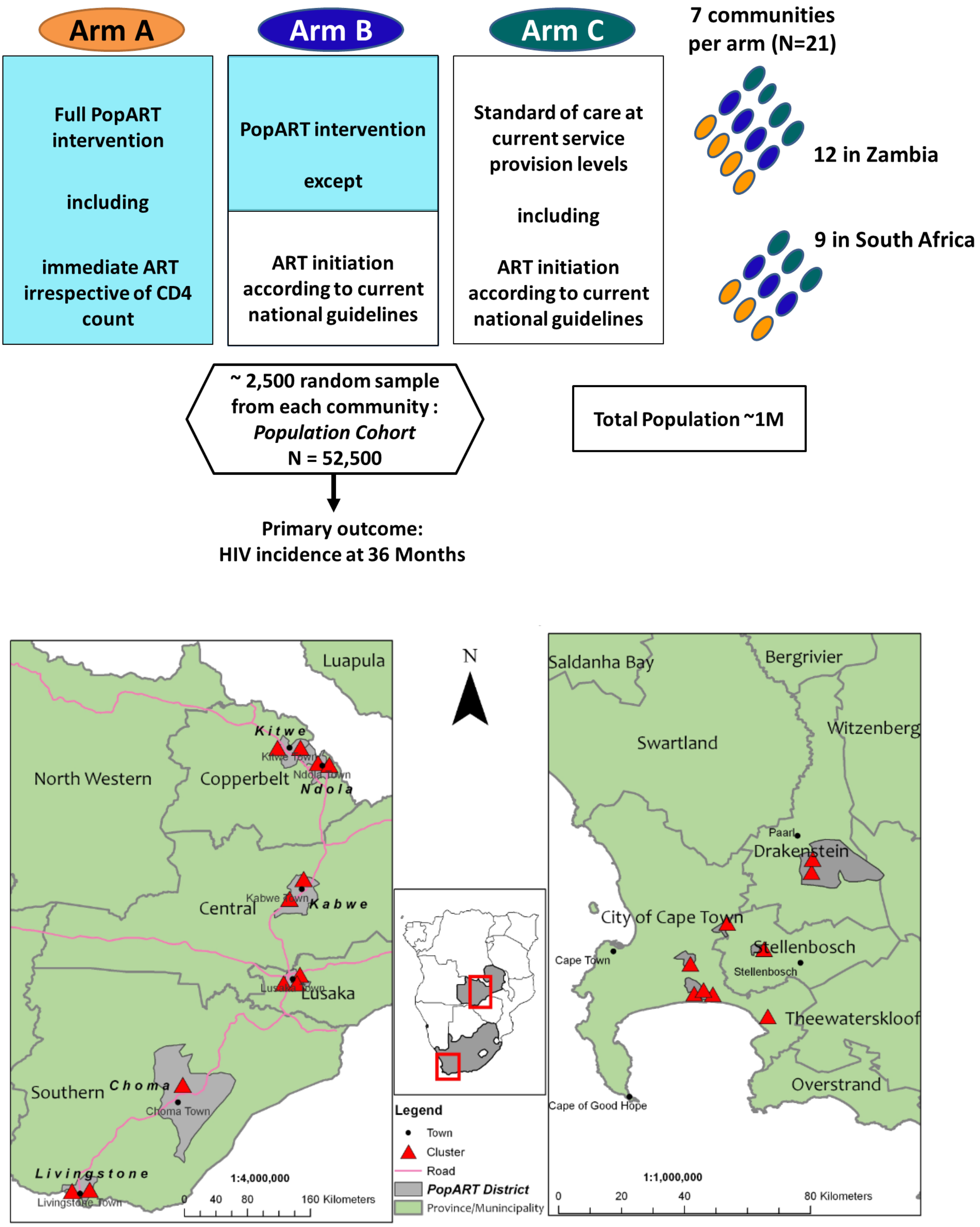

Legend: The upper half of the figure is a schematic diagram of the basic elements of the study design

as of the start of the trial. It illustrates the different levels of intervention offered in each of the three arms of the study, how the communities were divided between arms and between countries, and how the study outcome was measured. During study conduct, national guidelines changed to offer universal antiretroviral therapy (ART) to all HIV-positive individuals irrespective of CD4 count. The enrolled Population Cohort (evaluation cohort) was smaller than the target number, averaging approximately 2,300 per community (total 48,301).

The lower figure maps the locations of the communities (clusters) that participated in the trial in the Copperbelt, Central, Lusaka and Southern provinces of Zambia and the Western Cape province of South Africa.

Figure S2 - PopART combination prevention package and household intervention

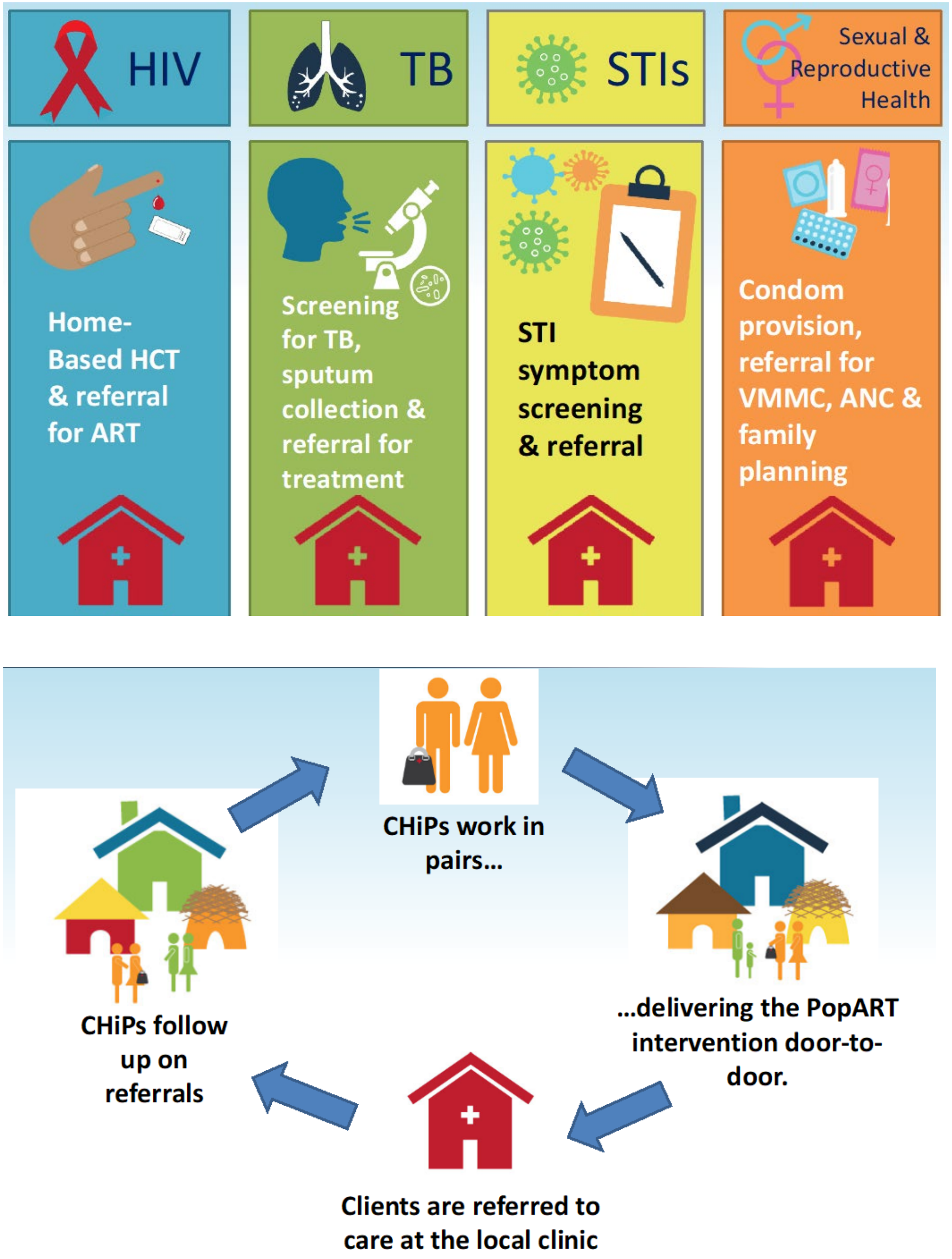

Legend: The upper half of the figure illustrates the components of the combination prevention package delivered at the household level. The lower half of the figure illustrates how the community healthcare workers (CHiPs) carried out their work in the field. During an annual round of the intervention, CHiPs would visit their assigned households, enumerating household

members, offering screening/testing services and providing referrals for care. CHiPs would return to the households some days later to follow up on referrals made, or to offer services to household members absent at the prior visit or to support clients on therapy to remain adherent. Although for some households the CHiPs would only make one visit per annual round, for many households the CHiPs made multiple visits per round.

Figure S3 - Timeline of HPTN 071 (PopART) study showing annual intervention rounds (R1-R3), Population Cohort survey rounds (PC0-PC36), the primary analysis period and changes in ART initiation thresholds by country

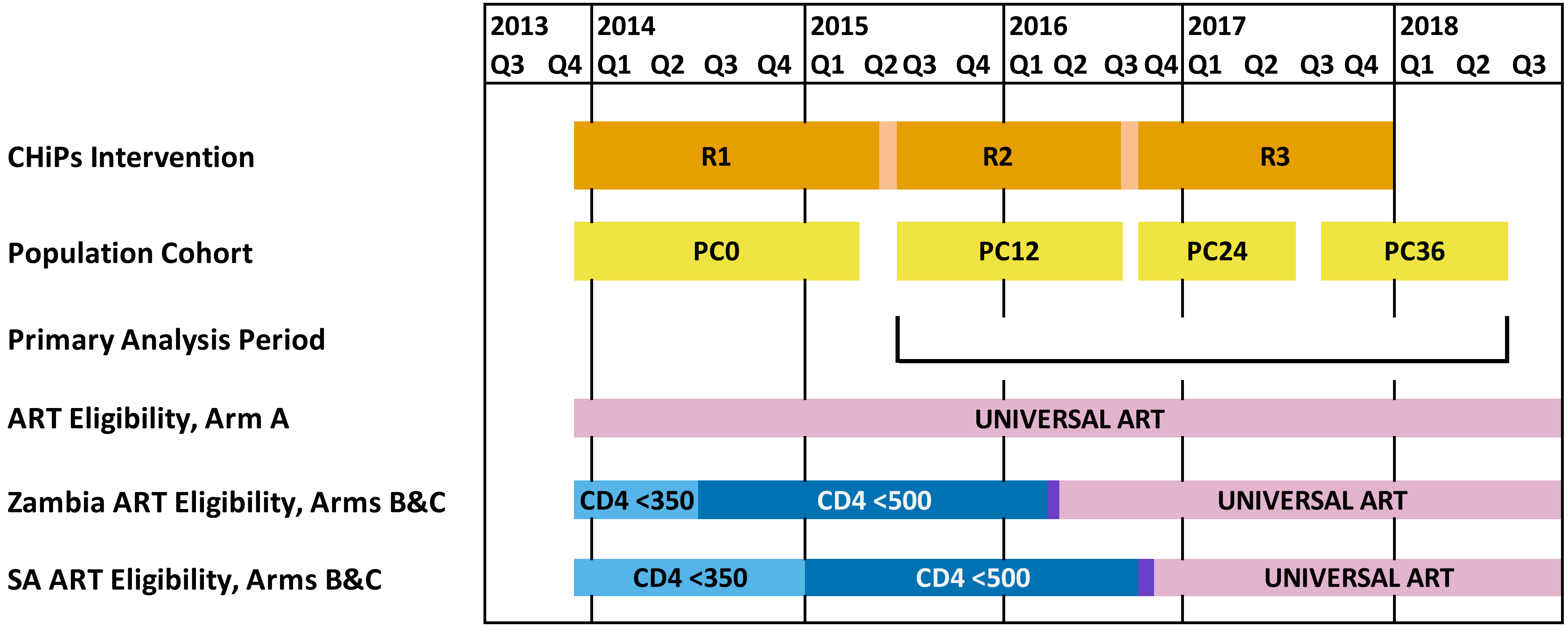

Legend: The dates shown for the start of **universal** ART (ART irrespective of a patient's CD4 cell count or WHO statge) refer to when this was implemented in study clinics in the respective countries. In Zambia, the first study clinic transitioned 19 April 2016 and the last 09 May 2016. This transition is represented by the dark purple band in the figure. In South Africa, the first study clinics transitioned 10 October 2016 and the last on 21 November 2016, a

period represented by the dark purple band in the figure. Study personnel, clinic staff and implementing partners worked to ensure that study clinics implemented the new policy as soon as local guidelines and logistics such as staff training would allow. Transition to universal ART in study clinics, therefore, often preceded transition in neighboring communiites.

Figure S4 - Screening and enrollment in PC0

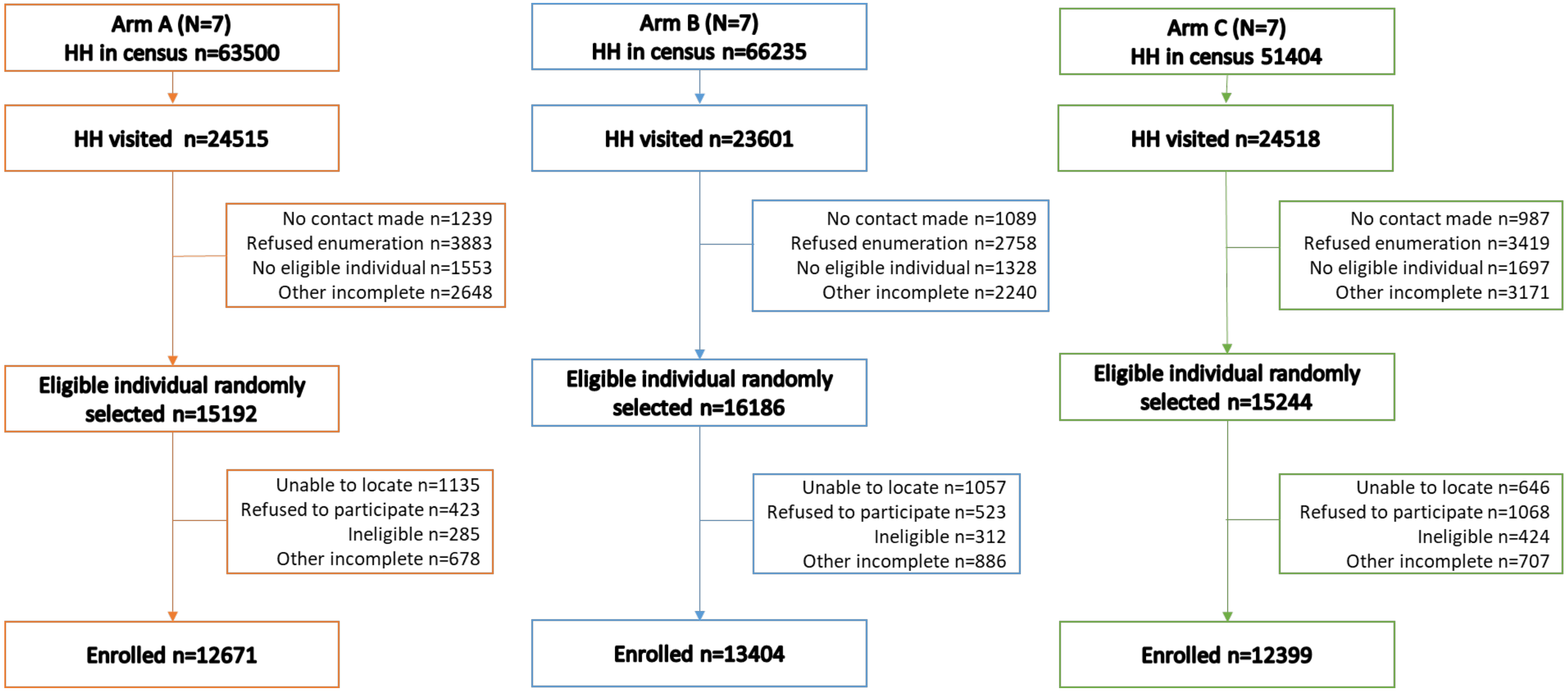

Legend: Prior to the study, a complete census of all households (HH) in the area defined as the study community was conducted to provide the sampling frame for the population cohort. Households were selected at random for participation, and researchers visited each household to obtain consent to enumerate all household members and select at random an age-eligible member. The selected member was then approached for full eligibility assessment and consent for participation in the population cohort.

Figure S5 - HIV incidence over study years in Arm C communities

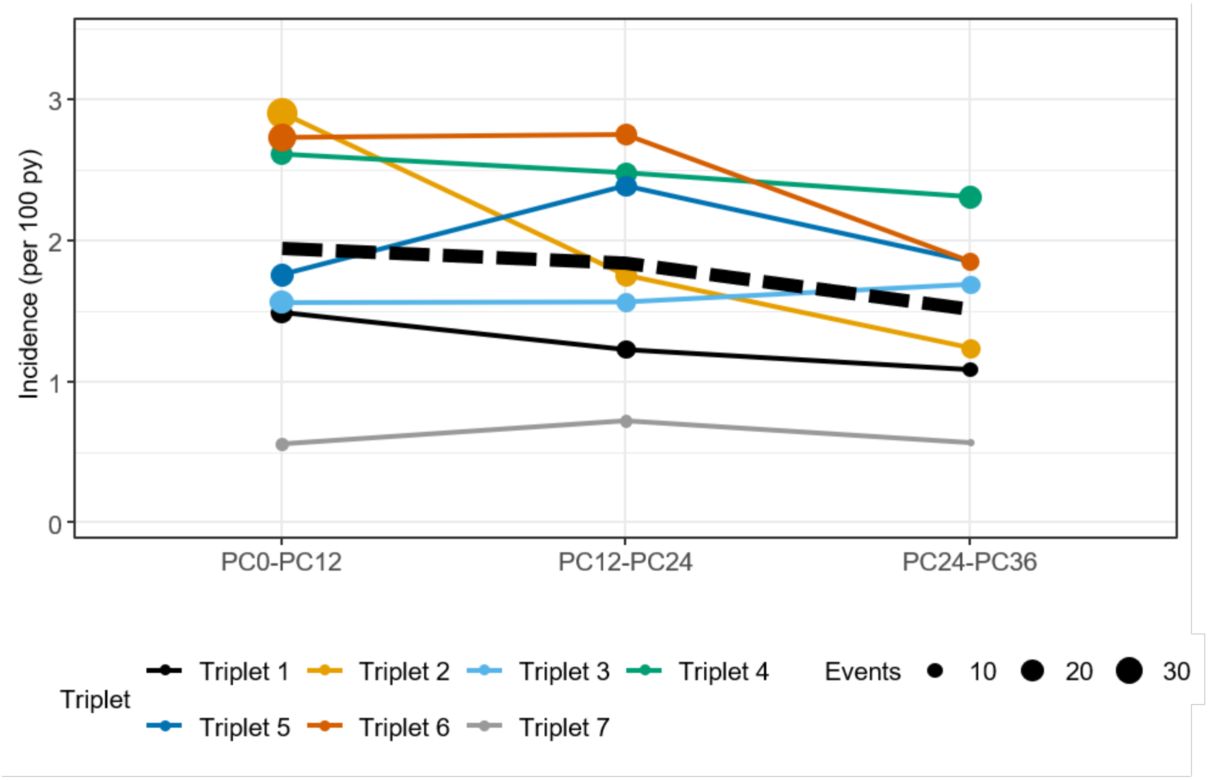

Legend: Incidence in each year is plotted over time. Mean incidence across communities in each period is shown by dashed black line. Using a Poisson regression model adjusting for triplet, we estimated 12.3% (95% CI 0.0%-22.9%) decrease in incidence each year. Colored lines represent each of the seven triplets (numbered 1 to 7). The size of the colored dots at the end of each line represents the number of events in each community.

Figure S6 - Estimated ART coverage at the end of the study, by triplet and study arm; estimated from the CHiPs data and extrapolated to total population aged ≥ 15 years

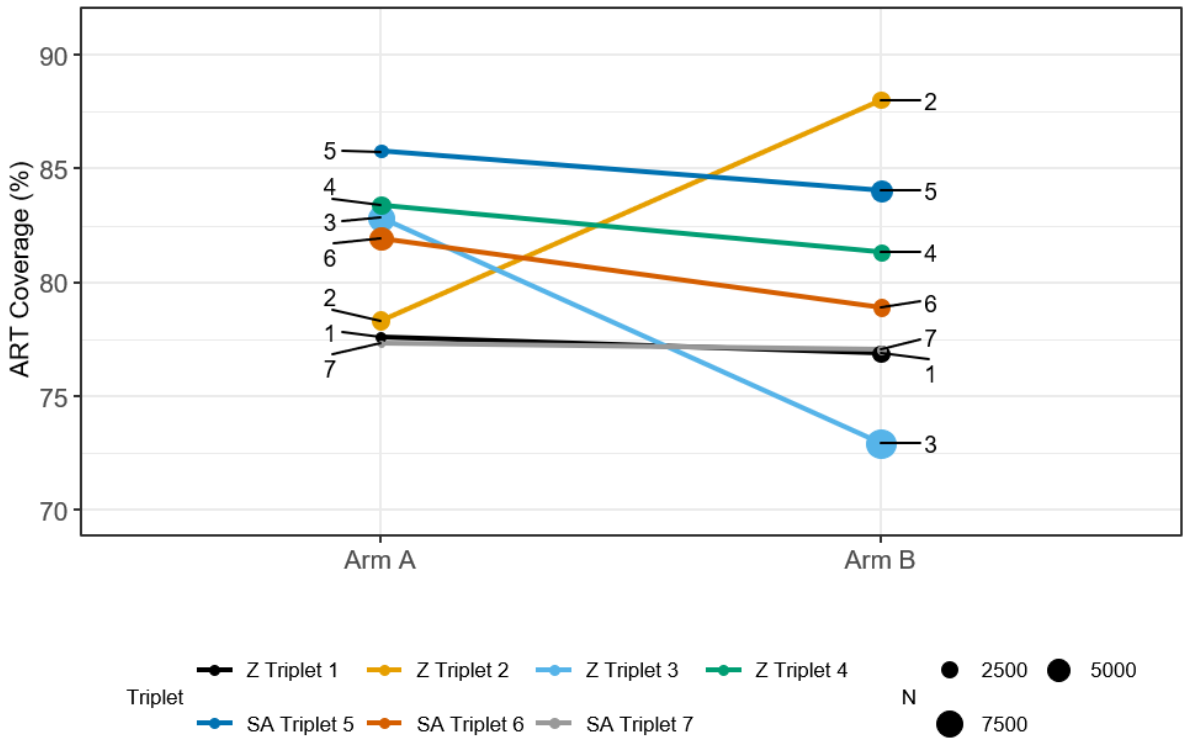

Legend: The plot shows the estimated proportion of HIV-positive people who reported that they were on ART in Arms A and B at the end of the study intervention period, based on the CHiPs data. Colored lines represent each of the seven triplets (numbered 1 to 7). The size of the colored dots at the end of each line represents the estimated number of HIV+ participants in each community.

Abbreviations: Z: Zambia; SA: South Africa

Table S1 - Number of eligible seroconverters and participants with missed visits affected by imputation.

Pattern of HIV assessment	PC0-PC24[1]	PC0-PC36[2]	PC12-PC36[3]
	No. events/Participants		
Arm A	21/603	20/197	25/556
Arm B	30/ 827	11/ 227	10/491
Arm C	16/ 618	15/270	22/613

[1] HIV negative at PC0; missing PC12 HIV status; HIV assessed at PC24

[2] HIV negative at PC0; missing PC12 and PC24 HIV status; HIV assessed at PC36

[3] HIV negative at PC12; missing PC24 HIV status; HIV assessed at PC36

Table S2 - Reasons for Termination by arm

	Arm A	Arm B	Arm C
**PC12 terminations**	2031	1639	1521
Refused further participation	416 (21%)	304 (19%)	228 (15%)
Relocated outside community	1357 (67%)	1026 (63%)	1117 (74%)
Incarcerated	6 (<1%)	8 (<1%)	12 (1%)
Incapacitated/in hospital	65 (3%)	141 (9%)	49 (3%)
Death	88 (4%)	81 (5%)	68 (4%)
Other	96 (5%)	70 (4%)	44 (3%)
**PC24 terminations**	1603	1608	1832
Refused further participation	413 (26%)	418 (26%)	406 (22%)
Relocated outside community	1013 (64%)	1005 (64%)	1217 (67%)
Incarcerated	7 (<1%)	12 (1%)	8 (<1%)
Incapacitated/in hospital	4 (<1%)	2 (<1%)	2 (<1%)
Death	52 (3%)	83 (5%)	72 (4%)
Other	106 (7%)	58 (4%)	117 (6%)
**PC36 terminations**[1]	3573	3330	3663
Refused further participation	625 (23%)	557 (22%)	848 (27%)
Relocated outside community	1801 (65%)	1742 (69%)	1990 (64%)
Incarcerated	8 (<1%)	16 (1%)	11 (<1%)
Incapacitated/in hospital	7 (<1%)	6 (<1%)	8 (<1%)
Death	68 (2%)	54 (2%)	67 (2%)
Other	266 (10%)	143 (6%)	170 (5%)

[1]At PC12 and PC24 participants who could not be located or refused visits were considered still in study follow-up and not terminated. At PC36, the study final visit, all participants who were not seen were terminated irrespective of reason

Table S3 - Effect of PopART intervention on HIV Incidence: subgroup analyses by age and sex.

Incidence (PC12-PC36)	Arm A	Arm B	Arm C	Adjusted RR[1]	Adjusted RR[1]
	No. of events/Total person years (rate per 100 py[2])			***Arm A vs Arm C***	***Arm B vs Arm C***
**Age 18-24**	81/4423 (1.75)	72/4590 (1.50)	75/4226 (1.72)	1.02 (0.79, 1.34)[3]	0.92 (0.70, 1.20)[4]
**Age 25+**	117/8576 (1.31)	85/9559 (0.83)	123/8337 (1.46)	0.90 (0.68, 1.20)[3]	0.58 (0.43, 0.76)[4]
**Men, age 18-24**	13/1438 (0.85)	10/1624 (0.56)	14/1645 (0.79)	1.19 (0.64, 2.21)	0.82 (0.44, 1.52)
**Women, age 18-24**	69/2985 (2.20)	62/2965 (1.98)	61/2581 (2.22)	1.04 (0.75, 1.43)	0.95 (0.69, 1.31)
**Men, age 25+**	23/2327 (0.74)	13/2676 (0.39)	25/2470 (0.94)	0.78 (0.31, 1.98)	0.48 (0.19, 1.22)
**Women, age 25+**	93/6240 (1.46)	72/6883 (0.93)	98/5866 (1.61)	0.92 (0.64, 1.32)	0.59 (0.41, 0.85)

[1]Adjusted for baseline HIV prevalence (and gender for age subgroups)

[2]Overall rates are geometric means of individual community rates

[3]P value for interaction by age for Arm A vs C: p = 0.587

[4] P value for interaction by age for Arm B vs C: p = 0.044

Abbreviations: RR = Rate Ratio

The statistical methods used in the primary analysis are also used here. Number of events and person years include imputation for seroconverters who missed PC12 and PC24 visits.

Table S4 - Effect of PopART intervention on HIV Incidence for each study year: PC0-PC12, PC12-PC24, PC24-36.

	Arm A	Arm B	Arm C	Adjusted RR[2]	Adjusted RR[2]
	No. of events/Total person years (rate per 100py[1])			***Arm A vs Arm C***	***Arm B vs Arm C***
**PC0-PC12**	137/8442 (1.32)	131/9272 (1.36)	156/8490 (1.74)	0.76 (0.48, 1.20)	0.81 (0.51, 1.27)
**PC12-PC24**	104/6725 (1.47)	80/7872 (0.93)	109/6397 (1.69)	0.85 (0.62, 1.15)	0.55 (0.41, 0.75)
**PC24-PC36**	94/6265 (1.34)	77/6276 (1.14)	89/6166 (1.40)	0.97 (0.62, 1.52)	0.86 (0.55, 1.35)

[1]Overall rates are geometric means of individual community rates

[2]Adjusted for age, sex and baseline HIV prevalence

The statistical methods used in the primary analysis are also used here.

Table S5 - Effect of PopART intervention on HIV incidence for entire study period (PC0-PC36).

Outcome	Arm A	Arm B	Arm C
**HIV incidence rate (PC0-PC36)**	*No. of events/Total person years (rate per 100py)*		
Triplet 1	34/2350 (1.45)	27/2656 (1.02)	43/3329 (1.29)
Triplet 2	67/3508 (1.91)	51/4226 (1.21)	70/3523 (1.99)
Triplet 3	35/2724 (1.28)	45/2572 (1.75)	52/3251 (1.60)
Triplet 4	66/3173 (2.08)	39/2725 (1.43)	56/2278 (2.46)
Triplet 5	67/2795 (2.40)	67/3152 (2.13)	48/2440 (1.97)
Triplet 6	49/3122 (1.57)	39/3585 (1.09)	62/2485 (2.49)
Triplet 7	17/3766 (0.45)	20/4509 (0.44)	23/3755 (0.61)
Overall IR[1]	335/21438 (1.44)	288/23425 (1.18)	354/21062 (1.63)
**Overall**	**Arm A vs Arm C**	**Arm B vs Arm C**	
Unadjusted Rate Ratio (95% CI)	0.88 (0.70, 1.11)	0.72 (0.57, 0.91)	1
P value[2]	0.262	0.011	
Adjusted Rate Ratio[3] (95% CI)	0.88 (0.68, 1.13)	0.74 (0.58, 0.96)	1
P value[4]	0.288	0.027	
**Men**			
Overall IR[1]	61/6282 (0.82)	45/7227 (0.59)	67/6928 (0.97)
Adjusted Rate Ratio (95% CI)	0.89 (0.51, 1.56)	0.66 (0.38, 1.14)	1
**Women**			
Overall IR[1]	274/15156 (1.68)	243/16198 (1.42)	287/14134 (1.90)
Adjusted Rate Ratio (95% CI)	0.90 (0.67, 1.2)	0.77 (0.58, 1.03)	1
**Age 18-24**			

Overall IR[1]	130/7458 (1.59)	110/7828 (1.32)	135/7242 (1.78)
Adjusted Rate Ratio (95% CI)	0.93 (0.73, 1.18)	0.82 (0.64, 1.04)	1
**Age 25+**			
Overall IR[1]	205/13980 (1.35)	178/15596 (1.11)	219/13820 (1.53)
Adjusted Rate Ratio (95% CI)	0.86 (0.63, 1.18)	0.71 (0.52, 0.97)	1

[1]Overall rates are geometric means of individual community rates

[2]P-value compared to the t-distribution with 12 degrees of freedom

[3]Adjusted for age, sex, baseline HIV prevalence

[4]P-value compared to the t-distribution with 11 degrees of freedom

Abbreviations: IR = incidence rate.

Imputation methods were not performed for PC0-PC36 summaries and analyses.

Table S6- Effect of PopART intervention on HIV incidence assessed in participants enrolled at PC0. Number of events and person years include imputation for seroconverters who missed PC12 and PC24 visits.

**Outcome**	**Arm A**	**Arm B**	**Arm C**
**HIV incidence rate (PC12-PC36)**	*No. of events/Total person years (rate per 100py)*		
Triplet 1	23/1096 (2.07)	11/1111 (0.96)	21/1763 (1.19)
Triplet 2	31/1773 (1.73)	29/2408 (1.20)	20/1592 (1.28)
Triplet 3	17/1145 (1.49)	16/1094 (1.45)	22/1586 (1.42)
Triplet 4	24/1356 (1.77)	14/1237 (1.14)	18/915 (1.99)
Triplet 5	35/1431 (2.41)	33/1811 (1.80)	26/1173 (2.22)
Triplet 6	24/1661 (1.44)	25/1991 (1.24)	30/1236 (2.41)
Triplet 7	13/1960 (0.64)	10/2488 (0.40)	14/2045 (0.70)
Overall IR[1]	165/10422 (1.54)	137/12140 (1.08)	152/10311 (1.49)
**Overall**	**Arm A vs Arm C**	**Arm B vs Arm C**	
Unadjusted Rate Ratio (95% CI)	1.04 (0.81, 1.34)	0.73 (0.57, 0.93)	1
P value[2]	0.74	0.017	
Adjusted Rate Ratio[3] (95% CI)	1.02 (0.78, 1.33)	0.74 (0.56, 0.96)	1
P value[4]	0.89	0.030	

[1]Overall rates are geometric means of individual community rates

[2]P-value compared to the t-distribution with 12 degrees of freedom

[3]Adjusted for age, sex, baseline HIV prevalence

[4]P-value compared to the t-distribution with 11 degrees of freedom

Abbreviations: IR = incidence rate.

Table S7 - Effect of PopART intervention on viral suppression at PC24: subgroup analyses by age and sex

PC24 Viral Suppression	Arm A	Arm B	Arm C	Adjusted PR[2]	Adjusted PR[2]
	# suppressed/no. HIV-positive (Overall prevalence[1])			***Arm A vs Arm C***	***Arm B vs Arm C***
**Age 18-24**	132/291 (45.8%)	107/218 (41.8%)	134/298 (42.7%)	1.06 (0.70, 1.61)[4]	0.94 (0.62, 1.43)[5]
**Age 25+**	1399/1868 (75.7%)	1211/1673 (70.5%)	1346/1885 (62.6%)	1.20 (0.96, 1.50)[4]	1.12 (0.90, 1.40)[5]
**Men, age 18-24[3]**	4/25 (14.5%)	9/17 (57.1%)	9/40 (18.0%)	Not estimable	Not estimable
**Women, age 18-24**	128/266 (48.9%)	98/201 (41.5%)	125/258 (47.5%)	1.03 (0.70, 1.52)	0.87 (0.59, 1.29)
**Men, age 25+**	179/269 (67.2%)	144/227 (61.5%)	170/290 (44.2%)	1.52 (0.86, 2.70)	1.39 (0.79, 2.47)
**Women, age 25+**	1220/1599 (77.0%)	1067/1446 (72.0%)	1176/1595 (68.5%)	1.12 (0.99, 1.28)	1.05 (0.93, 1.19)

[1]Overall prevalence is geometric mean of individual community prevalence

[2]Adjusted for age and sex,

[3] The number of HIV-positive men in age 18-24 was small in several communities, and some had zero with viral suppression, so log prevalence ratios were not defined and geometric means could not be computed

[4] P value for interaction by age for Arm A vs C: p = 0.597

[5] P value for interaction by age for Arm B vs C: p = 0.445

Abbreviations: PR = prevalence ratio

Table S8 - Viral suppression in HIV-positive PC participants at each study year.

	Arm A	Arm B	Arm C
	# suppressed/no. HIV-positive (Overall Prevalence[1])		
**PC0**	294/522 (56.0%)	300/525 (56.6%)	266/492 (50.4%)
**PC12**	379/529 (71.3%)	364/528 (68.4%)	313/491 (60.9%)
**PC24**	1467/1989 (74.8%)	1256/1725 (70.6%)	1421/2011 (63.8%)
**PC36**	371/490 (75.8%)	359/478 (74.3%)	332/450 (68.0%)

[1]Overall Prevalence is geometric mean of individual community prevalence

Viral suppression was assessed in a random sample of ~75 HIV-positive persons per community in PC0, PC12 and PC36. VL was assessed in all HIV-positive persons in PC24. Seroconverters are excluded.

Table S9 - Viral suppression at PC24 in HIV-positive PC participants who self-report current ART use.

	Arm A	Arm B	Arm C
	# suppressed /no. of self-reported HIV-positive participants on ART (Overall Prevalence[1])		
**PC0**	152/174 (85.9%)	189/207 (90.8%)	139/150 (84.8%)
**PC12**	234/263 (88.3%)	224/257 (86.2%)	178/197 (91.6%)
**PC24**	973/1096 (88.1%)	992/1119 (85.8%)	1066/1219 (83.2%)
**PC36**	228/261 (86.1%)	275/314 (86.7%)	245/279 (82.0%)

[1]Overall Prevalence is geometric mean of individual community prevalence

Viral suppression was assessed in a random sample of ~75 HIV-positive persons per community in PC0, PC12 and PC36, and in all HIV-positive persons in PC24. This table reports only those who self-reported they were HIV-positive.

Table S10 - ART coverage, and coverage against the first two of the UNAIDS 90-90-90 targets, at the end of the study, by study arm; estimated from CHiP data and extrapolated to total population aged ≥15 years

Indicator	Women		Men		Total	
	Arm A	Arm B	Arm A	Arm B	Arm A	Arm B
ART coverage: Proportion of HIV+ individuals who are on ART	84.6[1] (N=15,936)[2]	83.6 (N=17,586)	73.9 (N=8,388)	72.5 (8,948)	81.0 (N=24,324)	79.9 (N=26,534)
First 90: Proportion of HIV+ individuals who know their HIV+ status	94.1 (N=17,861)[3]	93.6 (N=19,807)	85.8 (N=9,332)	85.2 (N=10,082)	91.3 (N=27,193)	90.8 (N=29,889)
Second 90: Proportion of known HIV+ individuals who are on ART	89.9 (N=15,048)[4]	89.2 (N=16,392)	86.2 (N=7,257)	84.9 (N=7,468)	88.8 (N=22,305)	87.9 (N=23,860)

[1] All percentages are calculated as the average across 7 communities, i.e. the average of 7 community-specific values;

[2] N = estimated total number of HIV+ individuals who were resident in the community at the time that CHIPs first visited their household during the third (and last) annual round of intervention, and remained resident in the study community at the end of the study;

[3] N = estimated total number of HIV+ individuals who were resident in the community at the time that CHIPs first visited their household during the third (and last) annual round of intervention;

[4] N = estimated total number of HIV+ individuals who knew their HIV+ status, among HIV+ individuals who were resident in the community at the time that CHIPs first visited their household during the third (and last) annual round of intervention, and remained resident in the study community at the end of the study

REFERENCES

1. Seeley J, Bond V, Yang B, et al. Understanding the Time Needed to Link to Care and Start ART in Seven HPTN 071 (PopART) Study Communities in Zambia and South Africa. AIDS and behavior 2018.
2. Ministry of Health - Government of the Republic of Zambia. Adult and Adolescent Antiretroviral Therapy Protocols. https://wwwmedboxorg/zambia/adult-and-adolescent-antiretroviral-therapy-protocols/preview?q= 2010;Accessed 1st Feb 2019.
3. The Ministry of health and Community development Mother and Child Health. Zambia Consolidated Guidelines for Treatment and Prevention of HIV Infection. http://wwwnorthriseuniversitycom/wp-content/uploads/2017/07/Zambia-Consolidated-Guidelines-for-Treatment-and-Prevention-of-HIV-Infection-pdf 2013;Accessed 1st Feb 2019.
4. Ministry of Health - Government of the Republic of Zambia. Zambia Consolidated Guidelines for the Treatment and Prevention of HIV Infection. https://wwwmedboxorg/zambia/zambia-consolidated-guidelilnes-for-treamtent-prevention-of-hiv-infection/preview 2016;Accessed 1st Feb 2019.
5. National Department of Health - South Africa. The South African Antiretroviral Treatment Guidelines. https://sahivsocorg/Files/2013%20ART%20Treatment%20Guidelines%20Final%2025%20March%202013%20correctedpdf 2013;Accessed 1st Feb 2019.
6. National Department of Health - South Africa. National consolidated guidelines for the prevention of mother-to-child transmission of HIV (PMTCT) and the management of HIV in children, adolescents and adults. https://sahivsocorg/Files/ART%20Guidelines%2015052015pdf 2015;Accessed 1st Feb 2019.
7. Western Cape Government. The Western Cape Consolidated Guidelines for HIV Treatment: Prevention of Mother- to- Child Transmission of HIV (PMTCT), Children, Adolescents and Adults. https://wwwwesterncapegovza/sites/wwwwesterncapegovza/files/the-western-cape-consolidated-guidelines-for-hiv-treatment-2015-v26012016pdf 2015;Accessed 1st Feb 2019.
8. Hayes R, Floyd S, Schaap A, et al. A universal testing and treatment intervention to improve HIV control: One-year results from intervention communities in Zambia in the HPTN 071 (PopART) cluster-randomised trial. PLoS Med 2017;14:e1002292.
9. Floyd S, Ayles H, Schaap A, et al. Towards 90-90: Findings after two years of the HPTN 071 (PopART) cluster-randomized trial of a universal testing-and-treatment intervention in Zambia. PloS one 2018;13:e0197904.
